# Topical Collection “Pharmacology of Medicinal Plants”

**DOI:** 10.3390/biom11010101

**Published:** 2021-01-14

**Authors:** Barbara Romano, Giuseppe Lucariello, Raffaele Capasso

**Affiliations:** 1 Department of Pharmacy, School of Medicine and Surgery, University of Naples Federico II, 80138 Naples, Italy; barbara.romano@unina.it (B.R.); giuseppelucariello@hotmail.it (G.L.); 2 Department of Agricultural Science, University of Naples Federico II, 80138 Naples, Italy

The use of remedies based on medicinal plants continues to expand rapidly around the world, with many people now resorting to this type of product for the treatment and prevention of several pathologies. The past decade has seen a huge wave of acceptance and public interest in this area, with “natural” therapies gaining widespread use and becoming well consolidated in numerous countries. For such reasons, medicinal plants are currently the subject of great interest in research. Firstly, considering that of the approximately 400,000 species of existing botanicals, it is believed that less than 10% about have been studied for their biological activity, it is evident that the vegetable kingdom represents a source of molecules that are still largely unexplored, and therefore of great potential interest in drug discovery. There is so a demanding need to improve our knowledge regarding the pharmacological and biological activity of plant products. In this topic collection, we have collected 40 articles, summarized as below.

Zimmermann et al. in their study offer a procedure for making thapsigargin (Tg) and its derivatives more accessible in a sustainable way, facilitating the preparation of Tg prodrugs for the treatment of slowly developing cancer diseases like hepatocellular carcinoma and prostate cancer [1]. Azuama et al. have reported for the first time the anti-virulence activity of the medicinal plant *Azorella atacamensis* against the human opportunistic pathogen *Pseudomonas aeruginosa*. Interestingly, the mulinane-like diterpenoids putatively identified from *A. atacamensis* appear to be responsible for the observed virulence attenuation [2]. Fursenco et al. reviewed the evidence relating to *Solidago virgaurea*, a medicinal plant widely used in Europe and other parts of the world for its potential activities including its anti-inflammatory, analgesic, antifungal, antiparasite, cytotoxic, antitumor, antidiabetic, cardioprotective and antisenescence effects [3]. Hussain et al. have provided two potentially potent lead compounds—chalcone and anthraquinone, isolated from *Ranunculus muricatus*—which can be further developed for the design of novel and efficient drugs for the treatment of Alzheimer’s disease and type 2 diabetes, respectively [4]. Okon et al. evaluated the potential activity of magnoflorine (MGN), a quaternary aporphine alkaloid isolated from the roots of *Berberis cretica* L., as an anti-neoplastic therapy for lung, breast, glioma and rhabdomyosarcoma cancers, demonstrating that at high doses MGN inhibits proliferation, induces apoptosis and inhibits the cell cycle in S/G2 phases in a dose-dependent manner [5]. Mohamed et al. investigated the protective effect of linalool on renal function and tissues in a cisplatin-induced kidney injury rat model, with many mechanisms suggested for its activity. Linalool potentiated the cisplatin-cytotoxic effect, significantly reducing IC_50_, suggesting a reduction in cisplatin dose and consequently reducing side effects [6]. Utami et al. discussed the available evidence related to hydroxytyrosol (HT) activity in wound healing enhancement. HT is an essential molecule isolated from *Olea europea*, known for its antioxidative capacity. This review highlights the positive influence towards wound healing by the actions mediated by HT, becoming a bioactive compound highly recommended for wound care applications [7]. Kim et al. demonstrated that soybean embryo extract (SEE), which is a bioactive phytoestrogen, exerts anti-obesity effects through the activation of adipose tissue metabolism and exhibits a synergistic effect upon co-treatment with enzymatically modified isoquercitrin (EMIQ) [8].The study of Piatczak et al. examined the phenolic compounds in hydromethanolic extracts of *Salix alba* (L.) leaves and bark, as well as their antioxidant activity and cytotoxic potential. Leaf extract may be used as a potential new source of bioactive polyphenols with applications in cosmetics, and bark extracts can also be used but at lower concentrations [9]. Zahara et al. summarized information on the pharmacology, traditional uses, active constituents, safety and toxicity of *Holarrhena pubescens,* a medicinal plant extensively used in Ayurveda for treating various ailments such as rheumatism, leprosy, skin diseases, diarrhea, dysentery, etc. Some of its major bioactive components need further investigation to evaluate their potential as drug candidates [10]. Lammel et al. probed the potential to suppress the expression of the inflammatory markers shown by some natural remedies traditionally used against different inflammatory diseases, finding that among them the extract derived from the roots and rhizomes of *Peucedanum ostruthium* showed a pronounced and a dose-dependent inhibitory effect [11]. Leite et al. studied the chemical composition of the pulp of the Brazilian Savanna fruit *Dipteryx alata* and evaluated its toxic effects, its influence on the life expectancy of the nematode *Caenorhabditis elegans*, and its antioxidant activities in vitro and in vivo, demonstrating its potential use as a functional and nutraceutical food [12]. Lee et al. investigated whether coumarin ameliorated impaired bone turnover and remodeling under diabetic condition, finding that this positive effect from coumarin is mediated by suppressing the interaction between advanced glycation end product (AGE) and its receptor (RAGE) [13]. Lee et al. aimed to determine whether the gintonin-enriched fraction, a glycolipoprotein component of ginseng, has a preventive effect against obesity and found that it reduced lipid accumulation by reducing the expression of pro-adipogenic and lipogenic factors and increased lipolysis and thermogenesis, representing a potential treatment for obesity [14]. Panda et al. investigated the active principles of *Holigarna caustica* Oken that are responsible for its anthelmintic activity. The rationale of the study was based on its use by tribes of Northeast India for the treatment of intestinal problems, and the results of the study support the use of this plant for this this kind of problem [15]. Weber et al. evaluated the effects of a traditional herbal medicinal product containing myrrh (*Commiphora molmol* Engl.), coffee charcoal (*Coffea arabica* L.) and chamomile flower dry extract (*Matricaria chamomilla* L.) on the inflammatory crosstalk between immune and intestinal epithelial cells. The results provide a mechanistic basis for the use of the herbal combination of myrrh, coffee charcoal and chamomile flower extract in inflammatory bowel disease treatment and underline the potential benefits of the phytotherapeutic multi-component/multi-target approach in this complex pathogenesis [16]. Baldim et al. argued about the safety aspects of using some natural products and their several pharmacological properties that are attributed to ergot alkaloids as a result of their antibacterial, antiproliferative, and antioxidant effects, in particular for the treatment of glaucoma and the use of nanoparticles to improve the residence time of the particles in the eye, although further research is needed [17]. Alvarez-Collazo et al. studied whether the citrus flavanone hesperetin (HSP) has potentially beneficial effects on LQT3 syndromes (type 3 long QT syndromes), associated with arrhythmogenic gain of function mutations in the cardiac voltage-gated Na+ channel. Their conclusions were that HSP, despite its potential value for LQT3 treatment, is inadequate to treat some genetic variants [18]. Fecker et al. evaluated the phytochemical composition and biological activity of *Oenothera biennis* L. (OB) hydroalcoholic extract and aimed to provide directions for its antimicrobial effect, as well as its antiproliferative and pro-apoptotic potential against the A375 melanoma cell line, and its anti-angiogenic and anti-inflammatory capacity [19]. In their review, de Almeida Magalhães et al. performed a search for literature and technological patents aiming to treat and prevent diseases, as well as to prepare pharmaceutical formulations, suggesting a growing interest in *Euterpe oleracea* Mart. (EO) leaves, fruits and oil, with a set of actions pointing to the development of promising new drugs for clinical use, although more studies should be done to consolidate initial results [20]. Gall et al. revealed the effect of long-term cannabidiol (CBD) treatment on the chronic unpredictable mild stress (CUMS) model of depression. CBD exerted a prohedonic effect in rats subjected to CUMS, demonstrated by the increased sucrose preference after three weeks of treatment [21]. In their work, Vieira et al. studied the role of terpineol, a monoterpenoid alcohol, in depression. Molecular docking suggested that CB1 and CB2 receptors are the most promising targets of terpineol action, corroborated by the terpineol antidepressant-like modulation of CB1, CB2 and also D2-dopaminergic receptors [22]. Ali et al. investigated the reproductive actions of hookah smoke (HS) exposure on a murine model and the possible positive effects mediated by the prebiotic agent gum acacia (GA). Their work showed that concomitant administration of GA significantly mitigated all of the measured indices of oxidative and nitrosative stress and inflammation in the testicular homogenates from mice exposed to HS, which caused some adverse effects on the reproductive hormone levels in the plasma and on the indices of oxidative and nitrosative stress and inflammation in the testes. These results suggest that the intake of GA supplement may be recommended as a useful agent that can mitigate the adverse effect of tobacco use [23]. de Andrade de Carvalho et al. proposed a new solvent that accelerated the extraction method of Brazilian red propolis, a proposed new source of compounds with cytotoxic activity. To evaluate the cytotoxicity profile of the obtained bioactives, a cell viability assay was performed in different tumor cell lines. Most of the extracts exhibited moderate cytotoxic activity [24]. Lopes et al. studied the anti-inflammatory and antinociceptive effects of hydroalcoholic extracts from the *Machaerium hirtum* twig (HEMh), a medicinal plant commonly used in folk medicine to treat ulcers, cough and diarrhea, using in vivo experimental models. The study revealed the effect of HEMh action on TRPA1 receptors, associated with its effect on the opioid system, which is responsible for a diminished release of glutamate and NO, which partially explains this extract’s antinociceptive properties. Another important outcome of this study is the anti-inflammatory effect the extract exhibited, which is related to the inhibition of cyclooxygenase action [25]. Steroids are a pivotal class of hormones with a key role in growth modulation and signal transduction in multicellular organisms. Brassinosteroids (BRs) are a natural collection of phytosterols, which have structural similarity with animal steroids. Recent studies have indicated anticancerous, antiangiogenic, antiviral, antigenotoxic, antifungal and antibacterial bioactivities of BRs in animal test systems. In their review, Kohli et al. update this information with recent studies [26]. de Almeida et al. examined the anti-inflammatory and neuroprotective potential of the flavonoid agathisflavone (FAB), which is derived from the Brazilian plant *Poincianella pyramidalis*, in in vitro models of neuroinflammation. The major immunomodulatory effects on microglia and astrocytes are likely to be central to the neuroprotective action of FAB. Moreover, it may be a potential anti-inflammatory and neuroprotective agent to prevent and treat neuroinflammatory-related diseases [27]. Adnan et al. investigated innovative therapeutic effects of *Holigarna caustica* (Dennst.), a popular plant used in folk medicine in Bangladesh, which is often used by the local folk practitioner to treat a variety of chronic diseases. The methanol extract of *H. caustica* leaves (MEHC) has been proven to have promising anxiolytic and antidepressant efficacy. Additionally, further evidence of suppressing the release of inflammatory mediators indicates an anti-inflammatory potential, a preventive role in oxidative-stress-prompted anxiety and depression and a promising binding affinity towards various receptors in molecular docking analysis of this plant. Therefore, *H. caustica* can be considered a potential candidate for possible therapeutic intervention in neuropsychiatric disorders [28]. Sinan et al. attempted to elucidate the in vitro inhibitory action of *Piptadeniastrum africanum* stem bark (PA-SB) extracts on enzymes involved in the pathogenesis of complications such as type II diabetes, Alzheimer’s disease and skin hyperpigmentation disorders. Overall, data from the study contribute to the biological assessment of *P. africanum*, which appears to be a promising source of natural compounds with protective and neuromodulatory effects [29]. Liu et al. reviewed compounds of botanical origin and published since 2002. Most of them need further studies of their toxicity, mechanisms and structure–activity relationships to assess more fully their potential as drugs to control infections by intestinal parasitic nematodes. Medicinal plants hold great promise as a source of effective treatments, including anthelmintic therapy [30]. Athamneh et al. aimed to reveal the anti-colon cancer potential of *Origanum majorana* essential oil (OMEO) and its underlying mechanisms of action. This study demonstrated that OMEO exerts anti-colon cancer activity through activation of p38 MAPK signaling, induction of protective autophagy associated with downregulation of the mTOR/p70S6K pathway and activation of the extrinsic and intrinsic apoptotic pathway [31]. Nam et al. investigated the protective effects of gintonin, a novel ginseng-derived lysophosphatidic acid receptor ligand, that improves brain functions and protects neurons from oxidative stress, on the developing cerebellum after prenatal and postnatal Pb exposure. The study revealed the ameliorating effects of gintonin against Pb, suggesting the potential use of gintonin as a preventive agent in Pb poisoning during pregnancy and lactation [32]. Batiha et al. discussed the traditional uses, bioactive chemical constituents and pharmacological and toxicological activities of *Glycyrrhiza glabra* L. (licorice), a small perennial herb that has been traditionally used to treat many diseases, such as respiratory disorders, hyperdipsia, epilepsy, fever, sexual debility, paralysis, stomach ulcers, rheumatism, skin diseases, hemorrhagic diseases and jaundice [33]. The literature reports the importance of monoterpenes based on the extensive pharmacological action of this class, including wound healing and anti-ulcerogenic agents. Thus, Perico et al. reviewed the pharmacological actions and mechanism of action of monoterpenes used for peptic ulcer disease, providing the scientific basis for future translation in which the knowledge from preclinical research may be applied to the clinical practice of new therapies for this disease [34]. Desrosiers et al. studied the dried leaves of *Artemisia annua* (DLA) and pure artemisinin, which have been used for millennia to treat malaria. They showed that the distribution of artemisinin to several tissues and serum significantly increased when delivered as DLA in male and female rats. Furthermore, the data suggest that artemisinin is differentially eliminated in males compared to females and differentially metabolized from DLA vs. pure artemisinin. Overall, these results enhance our understanding of how artemisinin delivered from *A. annua* is more bioavailable than when delivered as a pure drug [35]. Batiha et al. reviewed traditional uses, bioactive chemical constituents and pharmacological and toxicological activities of *Syzygium aromaticum* L., a traditional spice that has been used for food preservation and possesses various pharmacological activities, rich in many phytochemicals as follows: sesquiterpenes, monoterpenes, hydrocarbon and phenolic compounds. Pharmacologically, cloves and their main constituents possess antimicrobial, antioxidant, anti-inflammatory, analgesic, anticancer and anesthetic effects. Moreover, they showed insecticidal, mosquito repellant, aphrodisiac, and antipyretic activities [36]. Nguyen et al. evaluated ethanol and aqueous extracts of *Adenosma bracteosum*, used in traditional and modern medicine in Vietnam for curing hepatitis, for their alpha-glucosidase inhibitory activities and anti-hyperglycemic effects on glucose-loaded hyperglycemic and streptozotocin-induced diabetic mice. The results obtained indicate that *A. bracteosum* has a great antidiabetic potential [37]. *Withania somnifera* Dunal (Ashwagandha) is a widely used medicinal herb in traditional medicinal systems, with extensive research having been carried out on various plant parts. Surprisingly, the seeds of *W. somnifera* have never been investigated for their therapeutic potential, so Balkrishna et al. aimed to investigate it. Here, they show that the fatty acids from *W. somnifera* seeds have strong anti-inflammatory properties, along with remarkable therapeutic potential on psoriasis-like skin etiologies [38]. In a review, Nugraha et al. investigated ethnopharmacological uses, chemical composition and biological activities of vascular epiphytic medicinal plants. These epiphytic medicinal plants are able to produce a range of secondary metabolites, including alkaloids, and a total of 842 phytochemicals have been identified to date. As many as 71 epiphytic medicinal plants were studied for their biological activities, showing promising pharmacological activities, including as anti-inflammatory, antimicrobial and anticancer agents [39]. Salehi et al. aimed to summarize the studies on biological effects of the avocado–soybean unsaponifiable (ASU) mixture, its chemical composition and pharmacotherapy, as well as applications in autoimmune, osteoarticular and menopausal disorders. In fact, characterized by its potent anti-inflammatory effects, the ASU mixture is recommended to act as an adjuvant treatment for osteoarthritic pain and as a slow-acting symptomatic treatment of hip and knee osteoarthritis, autoimmune diseases, diffuse scleroderma and scleroderma-like states [40].
